# ACT001 reduces the expression of PD-L1 by inhibiting the phosphorylation of STAT3 in glioblastoma

**DOI:** 10.7150/thno.41498

**Published:** 2020-05-01

**Authors:** Luqing Tong, Jiabo Li, Qiuying Li, Xuya Wang, Ravi Medikonda, Tianna Zhao, Tao Li, Haiwen Ma, Li Yi, Peidong Liu, Yang Xie, John Choi, Shengping Yu, Yu Lin, Jun Dong, Qiang Huang, Xun Jin, Michael Lim, Xuejun Yang

**Affiliations:** 1 Department of Neurosurgery, Tianjin Medical University General Hospital, Tianjin, China.; 2 State Key Laboratory of Medicinal Chemical Biology, College of Pharmacy, Nankai University, Tianjin, China.; 3 Department of Neurosurgery, Johns Hopkins University School of Medicine, Baltimore, MD, USA.; 4 Laboratory of Neuro-oncology, Tianjin Neurological Institute, Tianjin, China.; 5 Department of Neurosurgery, The Second Affiliated Hospital of Soochow University, Suzhou, China.; 6 Tianjin Medical University Cancer Institute and Hospital, Tianjin, China.; 7 National Clinical Research Center for Cancer, Tianjin, China.; 8 Key Laboratory of Cancer Prevention and Therapy, Tianjin, China.; 9 Tianjin's Clinical Research Center for Cancer, Tianjin, China.

**Keywords:** glioblastoma, ACT001, p-STAT3, PD-L1, immunosuppression

## Abstract

ACT001, which is derived from an ancient anti-inflammatory drug, has been shown to cross the blood-brain barrier in preclinical studies and has demonstrated anti-glioblastoma (GBM) activity in clinical trials. However, its pharmacological potential for anti-GBM immune response modulation remains unclear. The chemical structure of ACT001 indicates that it may bind to STAT3 and thus modulate antitumor immune response.

**Methods:** Bioinformatics and immunohistochemistry (IHC) were used to assess STAT3 and PD-L1 expression in gliomas. Western blotting, RT-PCR and immunofluorescence were used to detect PD-L1 and p-STAT3 expression in glioma cells exposed to ACT001. Chromatin immunoprecipitation, an ACT001-Biotin probe, and a dual-luciferase reporter assay were used to detect direct modulation. The *in vivo* efficacy of ACT001 was evaluated in GL261 murine glioma model. Survival analyses were conducted using the log-rank (Mantel-Cox) test.

**Results:** Bioinformatic analysis of 1,837 samples from 4 public glioma datasets showed that STAT3 mRNA expression was correlated with the degree of malignancy and therapeutic resistance and that STAT3 mRNA expression was related to immunosuppression, leukocyte infiltration, and PD-L1 expression. IHC staining of 53 tissue samples confirmed that relatively high phosphorylated STAT3 and PD-L1 protein expression was associated with a relatively advanced World Health Organization (WHO) glioma grade. Next, we confirmed that ACT001 treatment reduced PD-L1 expression and STAT3 phosphorylation. An ACT001-biotin probe was used to verify that ACT001 bound to STAT3. We also demonstrated that STAT3 bound to the PD-L1 promoter. The inhibition of PD-L1 expression and STAT3 phosphorylation by ACT001 could be rescued by STAT3 overexpression. Additionally, ACT001 inhibited GBM growth and decreased PD-L1 expression *in vivo*. The expression of the M2 markers CD206 and CD163 was decreased, while that of the antitumor immune markers iNOS and IFNγ was increased by ACT001 *in vivo*.

**Conclusion:** Our results demonstrate that STAT3 plays a key role in immunosuppression of glioma and is inhibited by ACT001. ACT001 inhibits PD-L1 transcription and modulates anti-tumor immune response in glioma bearing mice. These findings will help us to understand the mechanism of ACT001 in GBM therapy.

## Introduction

Glioblastoma (GBM) is a deadly disease with an overall median survival time of 14.6 months. Most patients have disease recurrence and eventually die with a short survival time. This outcome may be due to the unique immunosuppression in GBM. A large number of leukocytes have been reported to infiltrate in GBM and are related to a poor prognosis^1^. Immune checkpoints such as programmed death receptor 1 (PD-1), programmed death-ligand 1 (PD-L1) and T-cell immunoglobulin mucin receptor 3 (TIM3) are highly expressed in GBM tissue and correlate with poor outcome^2^-^4^. The brain seems to have a relatively immunosuppressive microenvironment. It has been reported that melanoma in the brain is more tolerogenic than melanoma in the flank or lungs^5^. Antigen-specific CD8 T cells are deleted and exhibit impaired cytotoxicity in the brain of melanoma bearing mice^5^. Therefore, immunotherapy for GBM needs to be further developed.

ACT001 is a promising drug for the treatment of GBM and it was designated as an orphan drug for GBM by the FDA. It is currently being evaluated in several clinical trials (ACTRN12616000228482, Australian New Zealand Clinical Trials Registry; ChiCTR-OIC-17013604, Chinese Clinical Trial Register). In a phase I dose-escalation study of ACT001 in patients with recurrent glioblastoma (rGBM), one rGBM patient developed a partial response (ongoing after 10+ months of treatment)^6^. This suggests that further investigation of the pharmacological mechanisms of ACT001 is needed. ACT001 is derived from the structural modification of parthenolide (PTL), a well-studied anti-inflammatory and anticancer agent^7^. However, PTL is unstable in both acidic and basic conditions, limiting its clinical application^8^. Micheliolide (MCL) has the same anticancer structure as PTL but has more persistent stability in the plasma than PTL^9^. Dimethylaminomicheliolide (DMAMCL) is the dimethylamino Michael adduct of MCL, and ACT001 is the fumarate salt form of DMAMCL. Both ACT001 and DMAMCL release MCL slowly and consistently, and both drugs diffuse through the blood-brain barrier (BBB)^9^-^11^. ACT001/DMAMCL have shown potent anticancer 11-13 and anti-inflammatory^14^-^16^ activity. PTL has been shown to significantly inhibit the activity of the NFκB and STAT3 pathways, which are important pathways in both inflammation and cancer^7^. However, how ACT001 regulates the anti-GBM immune response has not been elucidated.

Persistently phosphorylated STAT3 is observed in nearly 70% of human cancers^17^. STAT3 activation typically requires phosphorylation of the Tyr705 residue, resulting in STAT3 dimerization, nuclear translocation, and binding to a palindromic DNA consensus sequence. STAT3 is involved in epithelial-to-mesenchymal transition, proliferation, metastasis, cell cycle progression, stemness, and therapeutic resistance 18, 19. It has been hypothesized that suppressing STAT3 activation may elicit a robust antitumor immune response due to loss of immune tolerance and restoration of T cell responsiveness^20^. Previous studies have shown that activated STAT3 promotes PD-L1 transcription^21^, ^22^. STAT3 also participates in the modulation of macrophage polarization and T cell-mediated antitumor immune responses^23^. In fact, there is currently no comprehensive analysis of STAT3 expression in gliomas, particularly with respect to immunosuppression.

PD-L1 (encoded by CD274) mRNA expression is significantly associated with immunosuppression and predicts very poor survival in patients^3^. Chemoradiation increases PD-L1 expression^24^. Temozolomide (TMZ)-challenged GBM cells strongly suppress pro inflammatory activity via enhanced transcription of PD-L1 but no other immune checkpoints, such as CD276, HVEM or galectin-9^25^. STAT3 signaling is involved in TMZ-mediated PD-L1 induction^25^.

Overall, the STAT3-PD-L1 axis plays an important role in GBM pathogenesis, and we hypothesize that ACT001 may inhibit the progression of GBM through the inhibition of the STAT3-PD-L1 axis. In this study, we aimed to (1) characterize the expression of STAT3 in glioma and GBM; (2) elucidate the molecular mechanism of ACT001 with respect to STAT3 and PD-L1; and (3) determine the effect of ACT001 *in vivo* with respect to antitumor immune response.

## Methods

### Datasets

TCGA datasets were downloaded from UCSC Xena, and included GBM gene expression microarray (TCGAmic), GBM phenotypes, GBMLGG gene expression RNAseq (TCGAseq), and GBMLGG phenotypes. RNA sequencing of Diffuse Gliomas (CGGAseq) was downloaded from the CGGA website. The GSE16011 dataset was downloaded from Gene Expression Omnibus (GEO), and included Series Matrix File (GSE16011mic) and GPL8542. In total, data from 539 cases in TCGAmic, 702 cases in TCGAseq, 320 cases in CGGAseq, and 276 cases in GSE16011mic were used for analysis of STAT3 mRNA expression in gliomas.

In addition, GSE76384 was downloaded to study the effect of MCL on mRNA expression of PD-L1 and STAT3. GSE23806 was downloaded to analyze the PD-L1 expression in several cell lines under different growth conditions. RMA-normalized mRNA expression data was downloaded from the Cancer Cell Line Encyclopedia (CCLE) to analyze the PD-L1 expression in different cell lines.

### Patients and GBM tissues

Tissue microarrays and glioma pathologic diagnoses were obtained from the Department of Neurosurgery, Tianjin Medical University General Hospital, China, from August 2011 to April 2017^26^. Written informed consent was obtained from all donors or their relatives. The study was carried out in accordance with the principles of the Helsinki Declaration and approved by the ethical committee at Tianjin Medical University General Hospital. Each microarray consisted of 149 samples of intra-tumoral, tumor border, peri-tumoral tissue, or nontumor. For this project, we only analyzed the staining in intra-tumoral tissue or in nontumor tissue.

### Cells and cell culture

Human glioma cell lines U251MG, and TJ905 were purchased from the Chinese Academy of Sciences Cell Bank (China). Human glioma cell lines SNB19 and LN18 were purchased from the American Type Culture Collection (ATCC, US). The LNZ308 glioma cell line was generously provided by Prof. Huang of Tianjin Medical University General Hospital. Human glioma cell line TJ179 was isolated from human GBM tissue and cultured in nude mice following the protocol of Wang J et. al^27^. Mouse glioma cell line GL261 was generously provided by Prof. Yao in Fudan University. The HMO6 microglial cell line was purchased from Beijing Future Biotechnology Co. (China), Ltd. All glioma and microglia cell lines were cultured in Dulbecco's Modified Eagle's Medium (DMEM, Gibco, US) supplemented with 10% fetal bovine serum (FBS, Gibco, US). U937 monocytes were generously provided by Dr. Jin of Tianjin First Central Hospital and were cultured in Roswell Park Memorial Institute (RPMI) Medium 1640 (Gibco, US) supplemented with 10% fetal bovine serum (FBS, Gibco, USA). Phorbol 12-myristate 13-acetate (100 ng/ml, PMA, Solarbio, China) was added to stimulate U937 monocytes into macrophages. All cell culture media were supplemented with antibiotics (100 U/mL Penicillin-Streptomycin, Gibco, US) and incubated in 5% CO_2_ at 37 ℃.

### Antibodies

Antibodies against PD-L1 (A11273, dilution for western blot, 1:1000; dilution for IHC analysis, 1:100; dilution for IF analysis, 1:100) and STAT3 (A11867, dilution for western blot, 1:1000; dilution for IF analysis, 1:100) were obtained from ABclonal (Wuhan, China). Antibodies against CD163 (bs-2527R, dilution for IHC analysis, 1:100), CD206 (bs-4727R, dilution for IHC analysis, 1:100), iNOS (bs-2072R, dilution for IHC analysis, 1:100) and IFNγ (bs-0480R, dilution for IHC analysis, 1:100) were obtained from Bioss (Beijing, China). Antibodies against Phospho-STAT3 (Tyr705) (9145S, dilution for western blot, 1:1000; dilution for IHC analysis, 1:100; dilution for IF analysis, 1:100; dilution for ChIP, 1:100) were obtained from Cell Signaling Technology (USA). Antibodies against β-actin (TA-09, dilution for western blot, 1:2000), peroxidase-conjugated goat anti-rabbit IgG (ZB-2301, dilution for western blot, 1:5000) and Peroxidase-conjugated goat anti-mouse IgG (ZB-2305, dilution for western blot, 1:5000) were obtained from ZSGB‐BIO (Beijing, China). Alexa Fluor 594 donkey anti-rabbit IgG (A21207, dilution for IF analysis, 1:1000) and Alexa Fluor 488 donkey anti-mouse IgG (A21202, dilution for IF analysis, 1:1000) were obtained from Invitrogen (USA).

### ACT001, lentivirus and plasmids

ACT001 and ACT001-Biotin were supplied by Accenda Co., Ltd. (Tianjin, China) and stored at -20 ℃ until experimentation. ACT001 was diluted in distilled water for oral administration.

Lentivirus containing a STAT3 knockdown sequence (shRNA-STAT3) was generated in GV248. Its target sequence is 5'-CGGCAACAGATTGCCTGCATT-3' (GeneChem, China). Lentiviral transfection was performed according to the manufacturer's manual. After infection, cells were selected using 5 μg/ml puromycin solution.

Plasmids were purchased from Hanbio (China). A PD-L1 overexpressing plasmid was constructed using the pGL3 vector. A STAT3 overexpressing plasmid was constructed using the pcDNA3.1 vector. pRL-TK vector was used as an internal control for the dual luciferase reporter gene assay. Plasmids were transiently transfected into cells using Lipofectamine 3000 (Invitrogen, US).

### Immunohistochemical (IHC) and immunofluorescence (IF) staining

IHC and IF staining were conducted as previously described by Li et al^26^. Paraffin-embedded tissue was prepared for IHC staining. IHC staining was assessed by summing intensity and quantity scores. Intensity score was graded as 0 (negative), 1 (weakly positive, light brown), 2 (moderately positive, brown), or 3 (strongly positive, dark brown). Quantity score was graded as 0 (negative), 1 (≤25%), 2 (26-50%), 3 (51-75%), or 4 (>75%). ACT001-treated cells were prepared on microscope slides for IF staining. Images were obtained with a VANOX microscope (Olympus, Japan).

### Cell counting kit-8 assay (CCK8)

Cell viability with ACT001 treatment was estimated using CCK8 (CK04, DOJINDO, Beijing, China), according to the manufacturer's manual. Cells (2.5×10^3^ cells per well) were seeded for 24 h in 96-well plates and treated with ACT001 for 24, 48, 72, or 96 h. Then, the cells were incubated with CCK8 solution for 2 h, and absorbance was measured at 450 nm by using a microplate luminometer (BioTek, USA).

### Western blot and real-time PCR (RT-PCR) analysis

Western blot and RT-PCR analysis was carried out as previously described^28^. Before denaturation, total protein concentration was determined using a BCA Protein Assay Kit (PC0020, Solarbio, China) according to the manufacturer's instructions. 30 μg of each protein sample was analyzed. The primer sequences (Tianyihuiyuan, China) were as follows: PD-L1 Forward: 5′-CCTACTGGCATTTGCTGAACGCAT-3′, Reverse: 5′-ACCATAGCTGATCATGCAGCGGTA-3'; β-Actin Forward: 5′-ACCATTGGCAATGAGCGGT-3′, Reverse: 5′-GTCTTTGCGGATGTCCACGT-3′. Data were analyzed using the relative standard curve method and normalized to β-Actin.

### Pull-down of ACT001-biotin bound proteins

ACT001-biotin bound proteins were isolated as described previously by Li et al^29^. Briefly, cells were lysed, centrifuged, and the supernatant (1.5 mg/mL) was collected and equally divided into three samples. One supernatant sample was incubated with 100 μmol/L of ACT001-biotin and another sample was incubated with 100 μmol/L of ACT001-S-biotin in RIPA buffer overnight at 4 ℃. The third sample was used as input group. Then the samples were incubated with streptavidin beads, separated by SDS-PAGE, and visualized by silver staining and western blot. Our protocol for silver staining is described in detail in the supplemental section.

### Chromatin-immunoprecipitation (ChIP)

ChIP assay was performed using a ChIP Assay Kit (Cell Signaling Technology, US) as described by Huang et al^30^. Briefly, chromatin was crosslinked with 1% formaldehyde. Cells were lysed and sonicated with a Sonics Vibra-Cell processor (Sonics & Materials Inc., US). Chromatin was immuno-precipitated using the corresponding target protein antibody. Immuno-precipitated products were collected using Protein G agarose beads. DNA was purified with RNase A and Proteinase K. The PCR products were then electrophoresed on 2% agarose gels stained with GelRed. The following primers for the PD-L1 promoter were used for RT-PCR as recommended by Marzec et al.^22^: 5′- CAAGGTGCGTTCAGATGTTG -3′ and 5′- GGCGTTGGACTTTCCTGA- 3′. The ChIP protocol is described in detail in supplementary information.

### Dual-luciferase reporter assay

The dual-luciferase reporter assay was performed using the Dual-Luciferase® Reporter Assay System (Promega, China) according to the technical manual. Briefly, these plasmids were transfected into cells with lipofectamine 3000 (Thermo Fisher Scientific, US) for 3 days, and their signals were detected by a microplate luminometer (BioTek, US). The protocol of dual-luciferase reporter assay is described in detail in the supplementary information.

### Implantation and oral administration of ACT001

All animal experiments were approved by the Ethical Committee of the Tianjin Medical University General Hospital. *In vivo* experiments were performed using C57BL/6 immuno-competent mice (6 weeks old). Intracranial tumors were established by stereotactically implanting 5×10^5^ GL261 cells as described by Zeng et al.^31^. Tumor burden was monitored by luciferase imaging every week starting on day 7 after implantation, and the mice were randomly allocated into treatment arms after confirming tumor. Either 100 mg/kg or 400 mg/kg ACT001 or water was orally administrated every day starting on day 7 after implantation (Figure 8A). Overall survival of mice in all groups was monitored. Luciferin signal was detected with the *in vivo* imaging system (IVIS) every week. Tumor was considered eliminated when the signal of the tumor could not be detected compared to background. Tumor was considered stable if the tumor signal was less than 5 times greater than the day 7 and day 14 signal. Tumor progression was defined as a tumor signal that increased by more than five-fold from day 7 or day 14. The brains of the mice were carefully extracted when mice died or on day 42. These brains were fixed in 10% formalin and embedded in paraffin for IHC staining.

### Statistics

mRNA expression was analyzed in GraphPad Prism 8.01. Overall survival (OS) of glioma patients was analyzed only when both survival information and mRNA expression data were available in the dataset. Survival analyses were conducted using the Log-rank (Mantel-Cox) test in GraphPad Prism 8.01, except for the GSE16011 dataset. This was analyzed on the R2 website. Heatmaps were downloaded from Morpheus, which is a versatile matrix visualization and analysis software. Z scores for all expression data were calculated and shown via heatmap. The R package of Estimation of STromal and Immune cells in MAlignant Tumors using Expression data (ESTIMATE) and an online tool of Cell-type Identification By Estimating Relative Subsets Of RNA Transcripts (CIBERSORT) were used to evaluate leukocyte infiltration. All experiments were performed at least three times. The quantitative data are expressed as mean ± standard deviation. The unpaired t-test was used to compare the means of two groups, and a two-tailed p value of < 0.05 was considered statistically significant.

## Results

### STAT3 mRNA is related to glioma malignancy and therapeutic resistance

**Figure 1:**
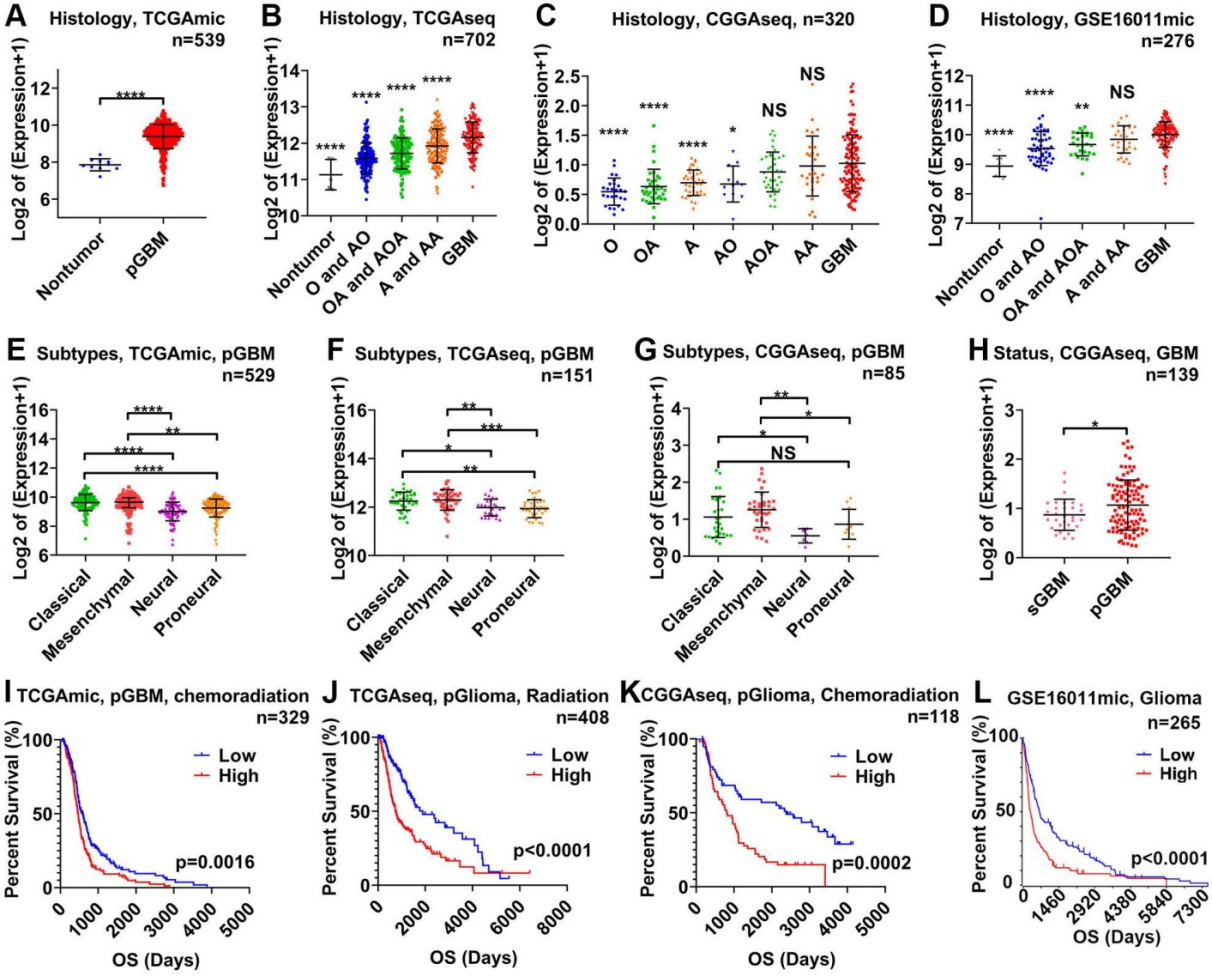
** STAT3 mRNA is related to the malignancy of glioma and therapeutic resistance.** STAT3 mRNA expression in nontumor tissue versus glioma tissue of varying WHO grades in the TCGAmic (A), TCGAseq (B), CGGAseq (C), and GSE16011 (D) datasets. The types of glioma evaluated included astrocytoma (A), oligodendroastrocytoma (OA), oligodendroglioma (O), anaplastic astrocytoma (AA), anaplastic oligodendroastrocytoma (AOA), anaplastic oligodendroglioma (AO) and primary GBM (pGBM). STAT3 mRNA in pGBM of different subtypes in the TCGAmic (E), TCGAseq (F) and CGGAseq (G) datasets. H. STAT3 mRNA in secondary GBM (sGBM) and pGBM. Survival curves for glioma (L), pGBM treated with chemoradiation (I), primary glioma (pGlioma) with radiation (J) and pGlioma with chemoradiation (K) based on STAT3 mRNA levels. *, p<0.05; **, p<0.01; ***, p<0.001; ****, p<0.0001; NS, not significant.

First, we analyzed the relationship between STAT3 mRNA and malignancy on the basis of World Health Organization (WHO) grade, GBM subtype, and GBM status using 1,837 samples from 4 glioma datasets. STAT3 mRNA expression was the highest in GBM tissue and lowest in nontumor tissue (Figure 1A-D). STAT3 mRNA expression was higher in astrocytoma (A) than in oligodendroglioma (O, Figure 1C). STAT3 mRNA expression in WHO grade III gliomas (including anaplastic astrocytoma (AA), anaplastic oligodendroastrocytoma (AOA) and anaplastic oligodendroglioma (AO)) was higher than that in WHO grade II gliomas (including astrocytoma (A), oligodendroastrocytoma (OA), and oligodendroglioma (O)) (Figure 1C). Given that the prognosis of the mesenchymal and classical GBM subtype are considered to be worse than that of other subtypes^32^, ^33^, STAT3 mRNA expression in the mesenchymal and classical GBM subtypes was higher than that in the neural and proneural GBM subtypes (Figure 1E-G). Furthermore, the prognosis of secondary GBM (sGBM) is reported to be better than that of primary GBM (pGBM), and STAT3 mRNA expression in sGBM was lower than that in pGBM (Figure 1H). According to clinical information from datasets, STAT3 mRNA expression was inversely related to overall survival in glioma patients (Figure 1I-L). Importantly, STAT3 mRNA expression was related to chemoradiation resistance in pGBM (Figure 1I) and primary glioma (pGlioma, Figure 1K).

**Figure 2:**
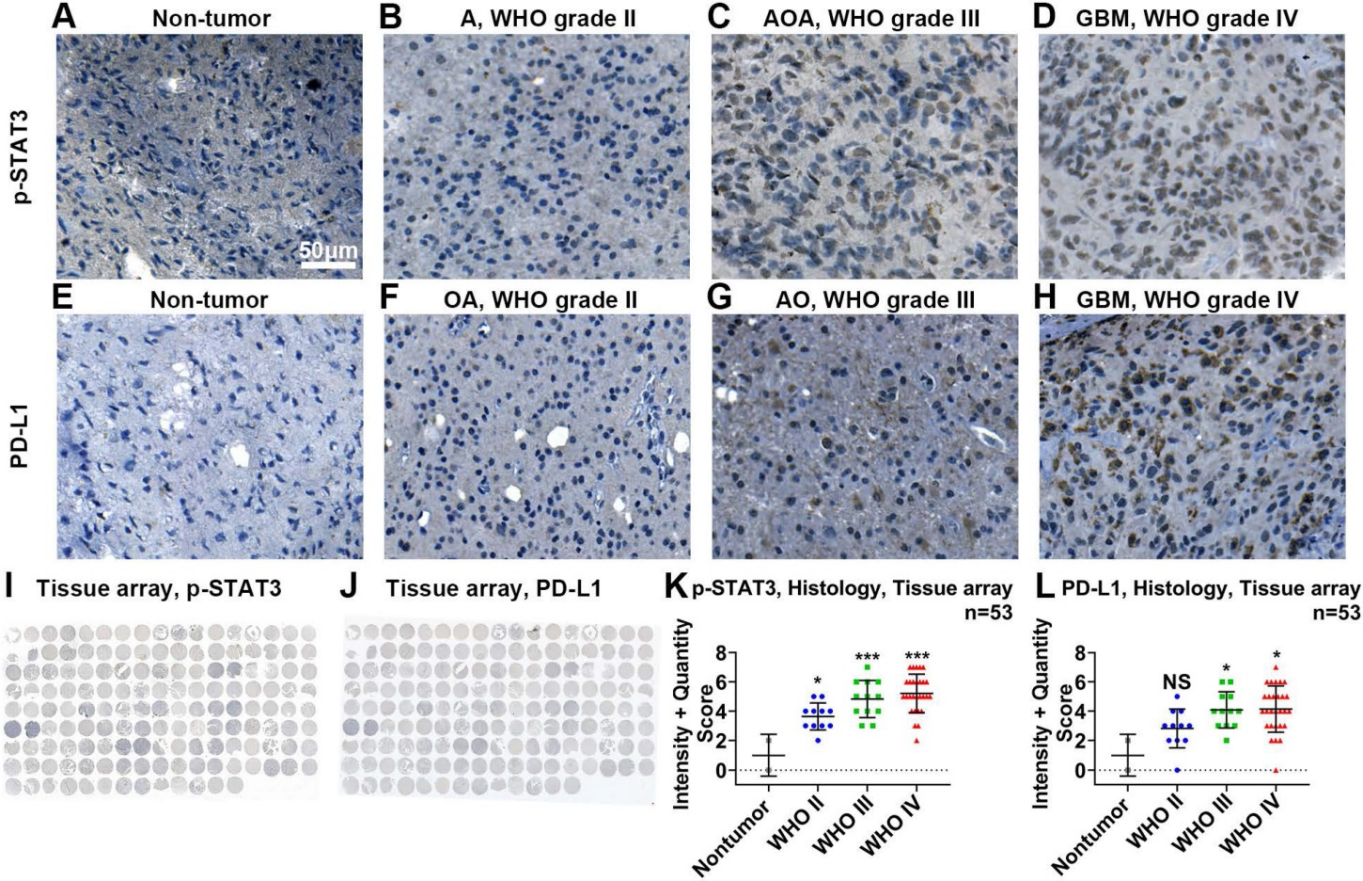
** p-STAT3 and PD-L1 protein levels are positively correlated with WHO glioma grades.** Representative images of p-STAT3 (Tyr705) expressed on nontumor (A), astrocytoma (B, abbreviated as A), anaplastic oligodendroastrocytoma (C, AOA) and glioblastoma (D, GBM) tissue samples. Representative images of PD-L1 expressed on nontumor (E), oligodendroastrocytoma (F, OA), anaplastic oligodendroglioma (G, AO) and glioblastoma (H, GBM). I and J show images of entire tissue microarrays. K and L. Nontumor tissue exhibited the lowest protein expression of p-STAT3 and PD-L1. P-STAT3 and PD-L1 protein levels positively correlated with WHO glioma grades. *, p<0.05; ***, p<0.001; NS, not significant.

Using IHC in tissue microarrays (Figure 2I), we found that the protein expression of p-STAT3 was related to malignancy classified by the WHO system (Figure 2A-D, K). The expression of p-STAT3 was hardly detected in nontumor brain tissue (Figure 2A) and WHO grade II glioma tissue (Figure 2B), while it was abundant in WHO grade III glioma tissue (Figure 2C) and GBM tissue (Figure 2D).

### STAT3 mRNA is related to immunosuppression and leukocyte infiltration in glioma

**Figure 3:**
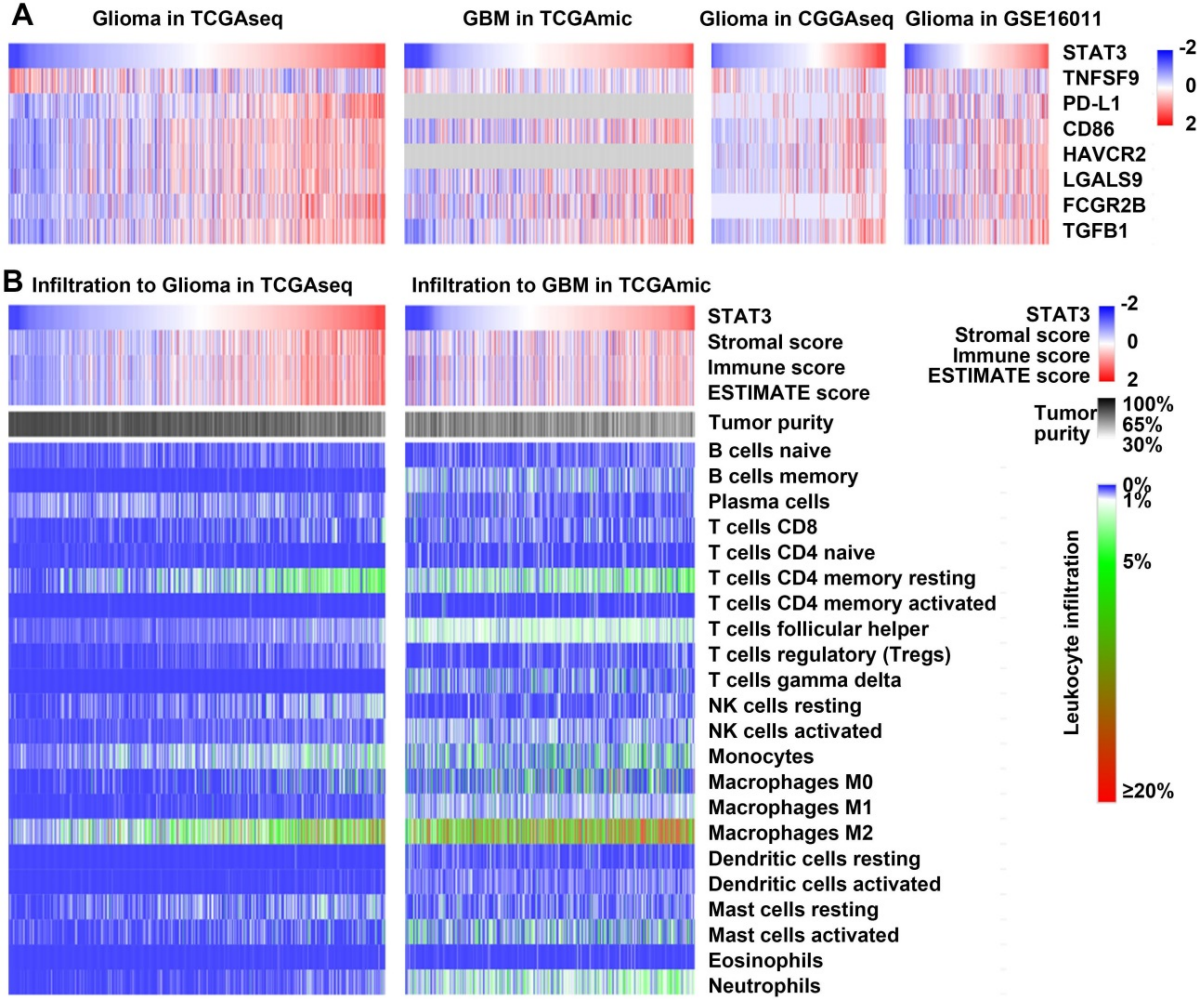
** STAT3 is related to immunosuppression and leukocyte infiltration in glioma.** A. Heat maps correlating the expression of STAT3 mRNA and T cell coinhibitory molecules (PD-L1, CD86, HAVCR2, and LGALS9), the immune risk gene FCGR2B, and the immunosuppressive cytokine TGFβ in the TCGAmic, TCGAseq, CGGAseq, and GSE16011 datasets. STAT3 mRNA expression inversely correlated with that of the endogenous costimulatory molecule TNFSF9 (also named 4-1BBL). B. Heat maps correlating STAT3 mRNA expression with the immune score, tumor purity, and leukocytes infiltration in the TCGAmic and TCGAseq datasets.

To understand the role of STAT3 mRNA expression in glioma immune system interactions, we examined the correlations between STAT3 mRNA and immune-related markers. STAT3 mRNA exhibited positive relations with T-cell coinhibitory molecules (PD-L1, CD86, HAVCR2, and LGALS9)^34^, immune risk genes such as FCGR2B^35^, and immunosuppressive cytokines such as TGFβ^36^. In addition, STAT3 mRNA exhibited an inverse relationship with TNFSF9 (also named 4-1BBL), which is known to be an endogenous costimulatory molecule (Figure 3A).

Nontumor cell infiltration has been shown to contribute to the immunosuppressive microenvironment in glioma^1^. The relationship between STAT3 mRNA and the infiltrating nontumor cells in glioma was characterized using the ESTIMATE algorithm developed by Yoshihara et al.^37^. The immune score was calculated by ESTIMATE to predict the level of infiltrating immune cells. STAT3 mRNA was inversely related to tumor purity and positively related to the immune score in both glioma and GBM datasets (Figure 3B). In addition, CIBERSORT, also known as in silico flow cytometry^38^, was used to further assess the relationships between STAT3 mRNA and 22 different immune cell populations. The results indicated that STAT3 mRNA was significantly correlated with M2 macrophage infiltration in glioma and GBM (Figure 3B). Overall, these results suggest that STAT3 plays an immunosuppressive role in the tumor microenvironment.

### ACT001 reduces the expression of p-STAT3 and PD-L1

Given that the activity of the STAT3 pathway mainly depends on activated STAT3 rather than STAT3 mRNA, we analyzed the correlations between PD-L1 and some genes related to STAT3 activation (mRNA transcripts of IL6, CLCF1, ICAM1, TGM2 and PDCD1LG2)^39^-^42^. We generated a heat map of mRNAs showing that PD-L1 mRNA expression was positively correlated with the mRNA expression of these genes (Figure S1A-C). In tissue microarrays (Figure 2J), we performed IHC and showed that PD- L1 was hardly detected in nontumor tissue (Figure 2E) and glioma tissue of WHO grade II (Figure 2F) but was abundant in glioma tissue of WHO grade III (Figure 2G) and GBM tissue (Figure 2H).

We found that PD-L1 mRNA expression could be reduced by treatment with MCL, which is the active form of ACT001 (Figure S1D). This suggests that ACT001 may reduce PD-L1 transcription.

Compared to glioma neurospheres and stem cell lines, conventional glioma cells exhibit higher levels of PD-L1 expression (Figure S2A). The mRNA expression of PD-L1 in SNB19, U251MG and TJ179 cell lines was higher than that in other cell lines (Figure S2B-D). Cell viability decreased when the concentration of ACT001 was higher than 10 μM, and the TJ179 cell line was most sensitive to high concentrations (40-80 μM) of ACT001 (Figure S2E-G). PD-L1 expression recovered on day 4 after ACT001 treatment (Figure S2H). Furthermore, the phenotype of glioma cells changed with ACT001 treatment, as seen when cultured tumor cells were observed under a light microscope (Figure S2I-K).

**Figure 4:**
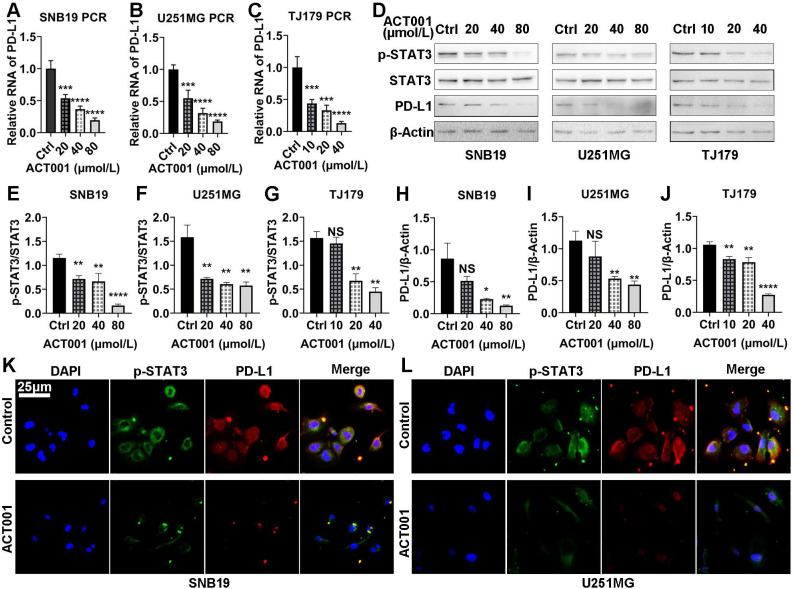
** Both p-STAT3 and PD-L1 levels are decreased by ACT001 treatment.** A-C. PD-L1 mRNA expression in glioma cells treated with ACT001 as detected by RT-PCR. D. PD-L1, STAT3 and p-STAT3 (Tyr705) protein levels in glioma cells treated with ACT001 as detected by western blotting. E-G. Relative p-STAT3 (Tyr705) protein level compared to the total STAT3 protein level. H-J. Relative PD-L1 protein level compared to that of β-Actin. K-L. IF of PD-L1 and p-STAT3 in glioma cell lines. *, p<0.05; **, p<0.01; ***, p<0.001; ****, p<0.0001; NS, not significant.

First, we studied the effect of ACT001 treatment on the mRNA and protein expression of PD-L1 and p-STAT3. At the mRNA level, RT-PCR results showed that PD-L1 expression was decreased significantly by ACT001 treatment in a dose-dependent manner (Figure 4A-C). At the protein level, the p-STAT3 level was decreased by ACT001 treatment whereas the STAT3 level did not vary significantly from that of β-Actin (Figure 4D-G). PD-L1 expression relative to the expression of the loading control β-Actin was decreased by ACT001 treatment, as observed by on western blotting (Figure 4D, H-J). Besides, when tumor cell lines were treated with 40 μM ACT001, both PD-L1 and p-STAT3 proteins were hardly detected by IF staining (Figure 4K, L). These results suggest that ACT001 decreases the expression of PD-L1 and phosphorylation of STAT3 in a dose-dependent manner.

### ACT001 decreases STAT3 activation and PD-L1 expression by directly binding to STAT3

**Figure 5:**
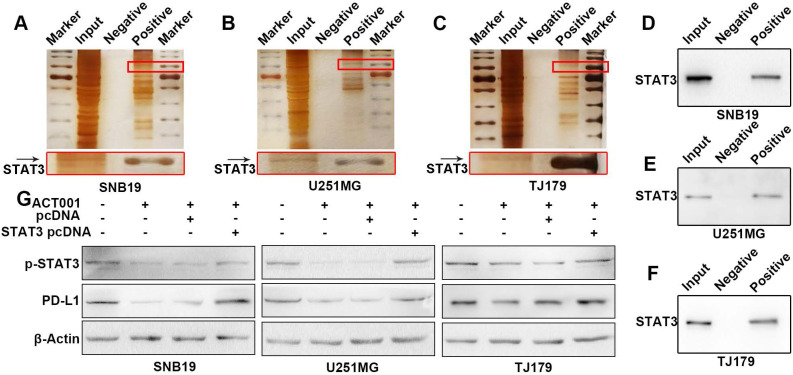
** ACT001 binds to the STAT3 phosphorylation site.** A-C. Glioma cell proteins in three different cell lines were detected via silver staining. Input refers to the whole protein lysate from the glioma cells. Negative refers to the ACT001-S-biotin probe solution. Positive refers to the proteins pulled down by the ACT001-biotin probe. D-F. Proteins of glioma cells were detected by western blotting using an anti-STAT3 primary antibody. G. PD-L1 and p-STAT3 (Tyr705) protein expression in glioma cells was detected by western blotting in different treatment arms. STAT3 pcDNA is a plasmid used to overexpress STAT3.

To further elucidate mechanism of the interaction between ACT001 and STAT3, proteins directly bound by ACT001 were pulled-down by using an ACT001-biotin probe. The silver staining results showed bands at approximately 100 kDa, which is similar to the molecular weight of STAT3 (Figure 5A-C). To confirm that ACT001 binds to STAT3, we utilized western blotting which showed that STAT3 was one of the proteins pulled down by the ACT001-biotin probe (Figure 5D-F). Furthermore, the reductions in PD-L1 and p-STAT3 level induced by ACT001 treatment were reversed by overexpression of STAT3 (Figure 5G). This further suggests that the pharmacological effects of ACT001 are dose dependent.

**Figure 6:**
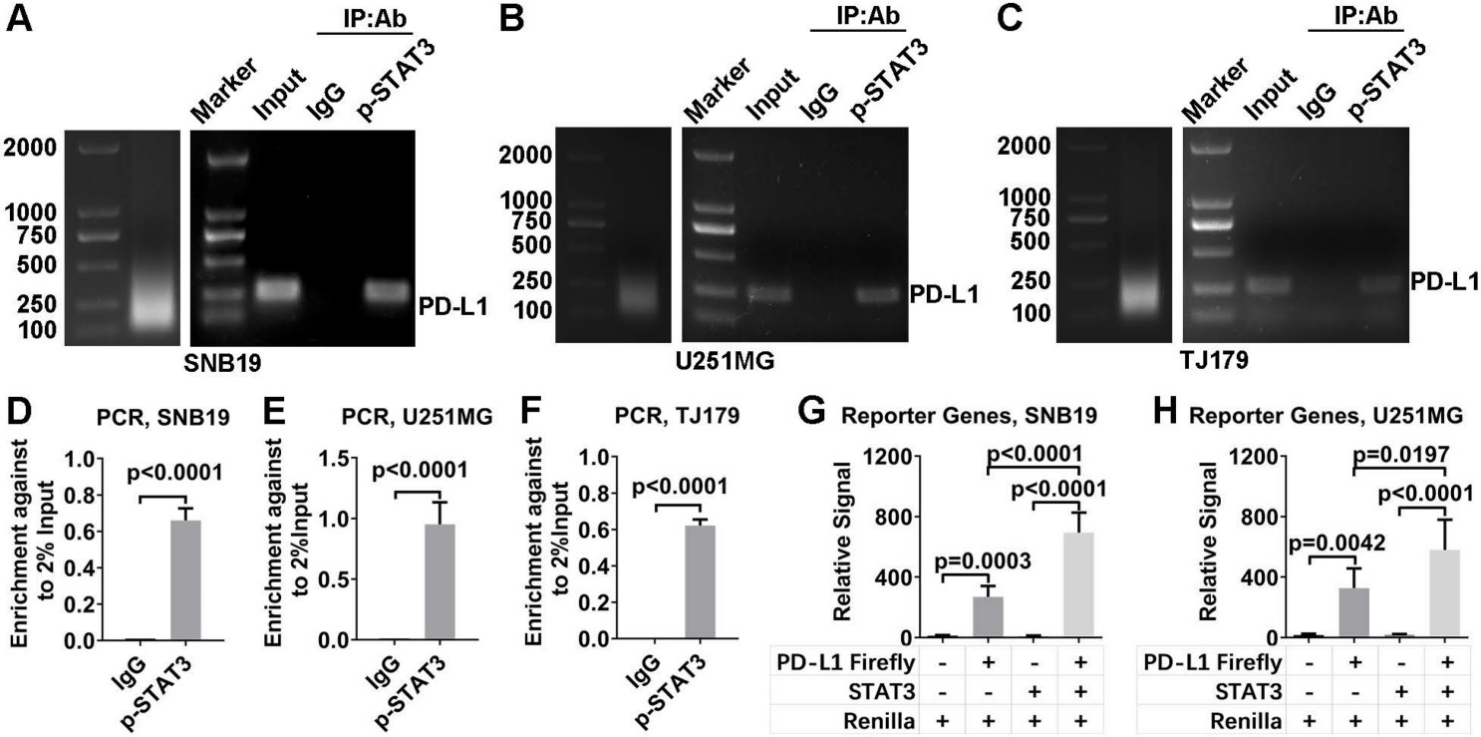
** p-STAT3 binds to the PD-L1 promoter.** A-C. ChIP was performed to verify that p-STAT3 binds to the PD-L1 promoter. The left lanes show the size of the ultrasonicated product. The right lanes show the PD-L1 promoter detected by agarose gel electrophoresis. Input refers to the whole lysate from glioma cells. The IgG group refers to the DNA pulled down by IgG. The p-STAT3 group refers to the DNA pulled down by p-STAT3. D-F. The PD-L1 promoter was PCR amplified. H, I. A dual-luciferase reporter assay was used to verify that PD-L1 transcription is promoted by STAT3. Vectors or overexpressing plasmids were transfected to glioma cells and the ratio of Firefly luciferase signal to Renilla luciferase signal was calculated.

Next, ChIP and dual-luciferase reporter assays were used to determine whether p-STAT3 promotes the transcription of PD-L1. DNA bound to p-STAT3 was pulled down in a ChIP assay. The DNA was PCR amplified with primers specific for PD-L1 promoter and electrophoresed in an agarose gel (Figure 6A-F). These results confirmed that STAT3 indeed demonstrated strong binding to the PD-L1 gene promoter. This finding was further supported by a dual-luciferase reporter assay, which showed that PD-L1 expression was increased by overexpressing STAT3 (Figure 6H, I). In addition, the PD-L1 protein level could be reduced by knocking down STAT3 expression and subsequently rescued by overexpressing STAT3 (Figure S1E and F). These results are consistent with the results of an electromobility shift assay (EMSA) performed with T cell lymphoma cells^22^.

**Figure 7:**
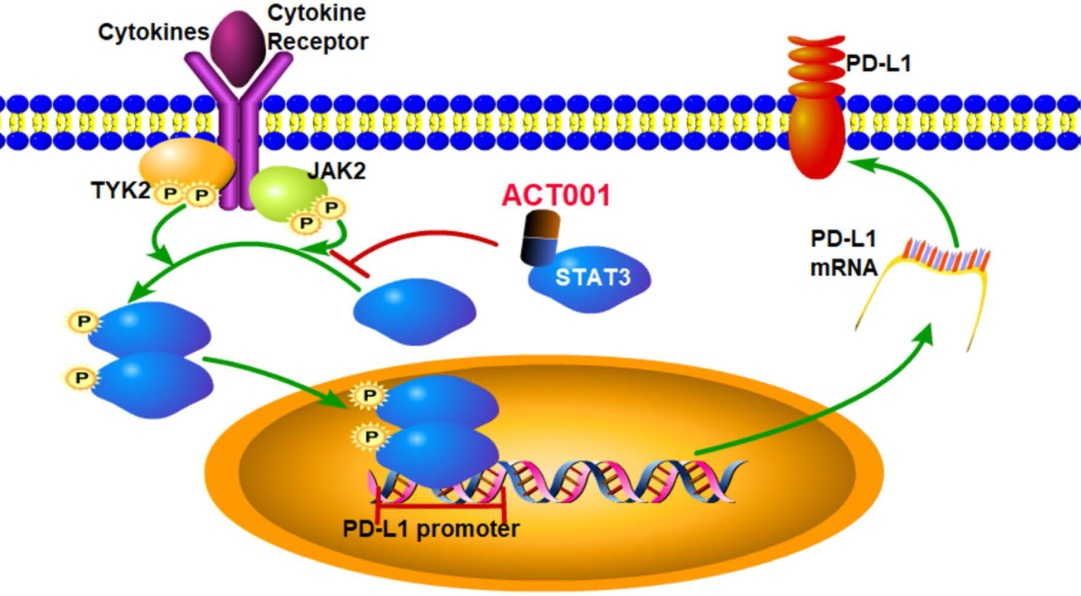
** Pharmacological mechanism of ACT001.** ACT001 directly binds to STAT3 and inhibits the phosphorylation of STAT3. PD-L1 transcription, which is promoted by p-STAT3 binding to the promoter of PD-L1, is decreased by ACT001 suppressing STAT3 phosphorylation.

Our results suggest the pharmacological mechanism of ACT001 (Figure 7). ACT001 directly binds to STAT3 in a dose dependent manner and inhibits the phosphorylation of STAT3. PD-L1 transcription, which is promoted by p-STAT3 binding to the promoter of PD-L1, is decreased by ACT001 inhibiting STAT3 phosphorylation.

### ACT001 decreases p-STAT3 and PD-L1 expression and suppresses the progression of glioma *in vivo*

**Figure 8:**
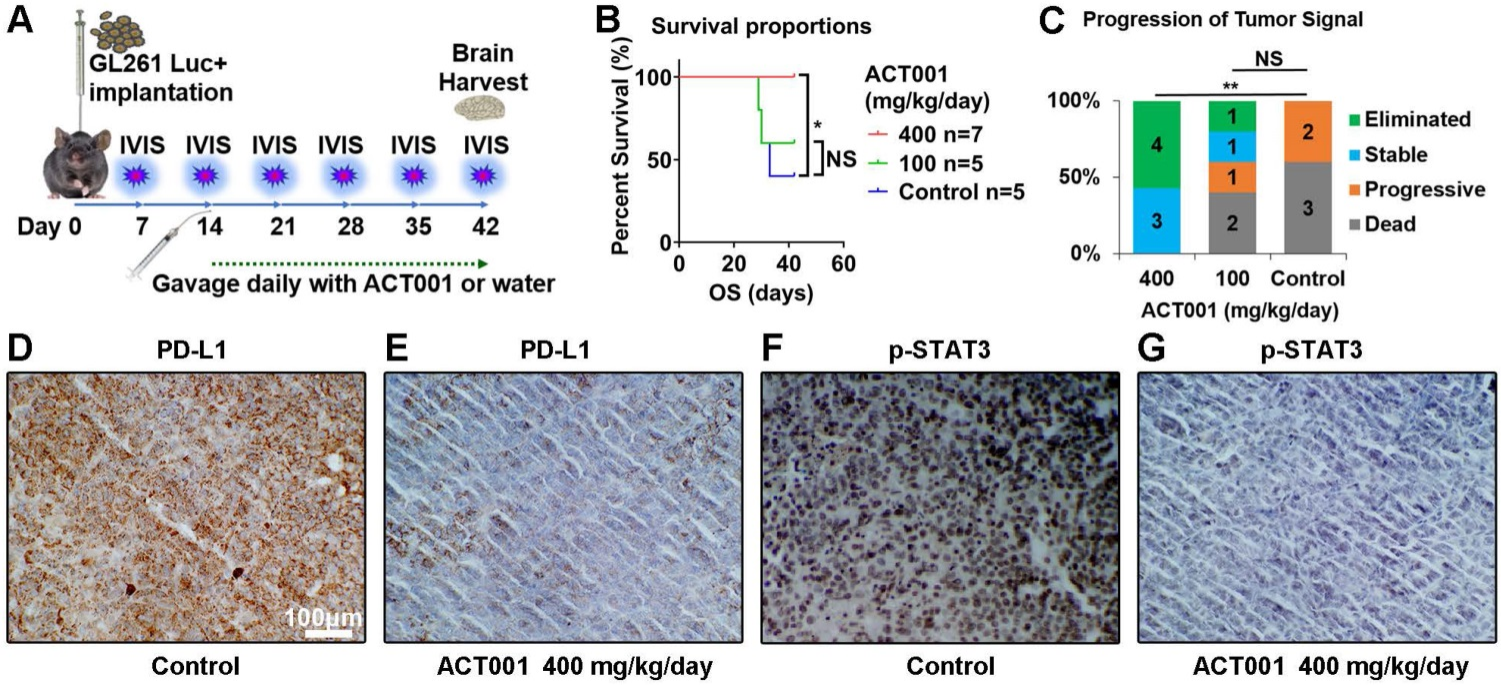
** ACT001 decreases p-STAT3 and PD-L1 expression and inhibits the progression of glioma *in vivo*.** A. Schematic diagram of implantation, luciferase imaging, and ACT001 administration. B. Survival curves of glioma bearing mice. The mice that remained alive at day 42 were sacrificed. C. Progression of tumor signals. Elimination of a tumor means that the signal for the tumor could not be detected compared to the background signal. Stable tumor means that the tumor signal was less than 5 times greater than the signals observed on day 7 and day 14. Progress is defined as a tumor signal that grows more than five-fold from the signals on days 7 and 14. PD-L1 (D and E) and p-STAT3 (F and G) were detected in tumor tissue from mice by IHC. *, p<0.05; **, p<0.01; NS, not significant.

Next, we validated the efficacy of ACT001 in prolonging survival by affecting the STAT3-PD-L1 axis in a murine glioma model. A GL261-C57BL/6 murine model was used due to the presence of a competent immune system (Figure 8A). With ACT001 treatment at a dose of 400 mg/kg/day, mice survived significantly longer than control mice (Figure 8B). In the 400 mg/kg/day treatment arm, tumors in 4 out of 7 mice were eliminated, and the other 3 mice did not experience tumor size progression (Figure 8C, Figure S3). In contrast, treatment with 100 mg/kg/day ACT001 failed to produce a significant survival benefit (Figure 8B, C). The mice used in this survival study were sacrificed on day 42, and tumor tissue was collected to detect PD-L1 and p-STAT3 by IHC (Figure 8D-G). Compared to the control arm, the 400 mg/kg/day ACT001 treatment arm showed decreased PD-L1 and p-STAT3 protein levels. The details for luciferase imaging and dynamic changes in tumor volume are listed in Figure S3.

### ACT001 decreases the infiltration of M2 macrophages and promotes the T cells response *in vivo*

**Figure 9:**
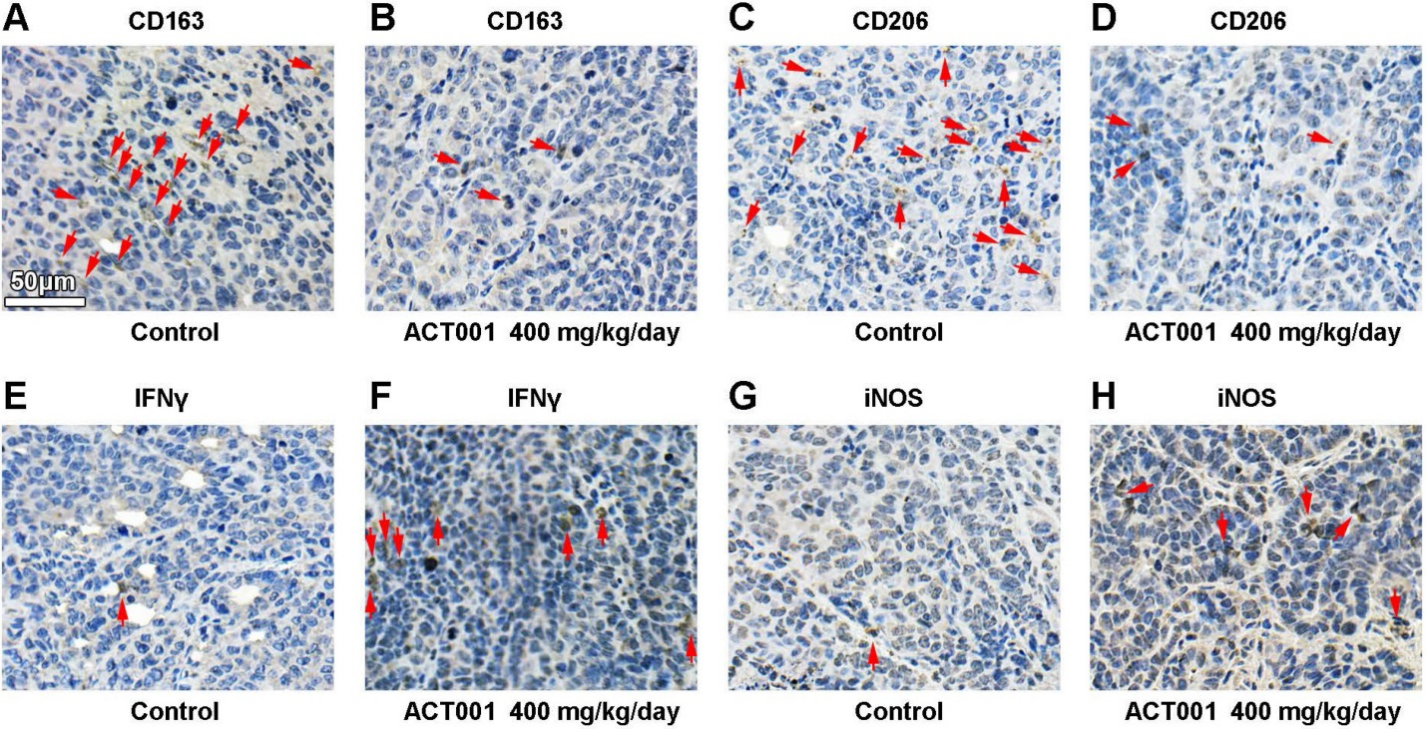
** ACT001 decreases M2 macrophage numbers and increases antitumor immune response *in vivo*.** Red arrows, positive stainings. M2 macrophages were stained for the markers CD163 (A and B) and CD206 (C and D). T cell-mediated cytotoxicity was assessed by IFNγ (E and F) staining. M1 macrophage mediated antitumor response was assessed by iNOS (G and H) staining.

Our bioinformatic analysis showed that STAT3 mRNA expression significantly correlated with M2 macrophage infiltration in glioma and GBM (Figure 3). CD163 and CD206 are well-accepted markers of M2 macrophages^43^. We detected CD163 and CD206 by IHC staining of GL261 glioma tissue samples and found that the levels of both markers were decreased after ACT001 treatment (Figure 9A-D).

Furthermore, we tested whether the inhibition of PD-L1 transcription by ACT001 increases the antitumor immune response. M1 macrophages convert arginine into nitric oxide (NO) through inducible nitric oxide synthase (iNOS), and cytotoxic T cells and NK cells secrete IFNγ to promote antitumor activity. Our results showed that both IFNγ and iNOS levels were increased in the ACT001 treated groups (Figure 9E-H).

## Discussion

STAT3-PD-L1 axis plays a key role in cancer immunosuppression. STAT3 is dispensable for the initial growth of carcinogenesis but it is critical for cancer immune evasion^44^. Tumor cells respond to activated T cells by activating STAT3^45^. Loss of STAT3 enhances T-cell recruitment and activation and a STAT3-dependent chronic inflammation is required for the cancer immune evasion^44^. The restrain of tumor progression by STAT3 inhibition was correlated with an M1 macrophages and accumulation of NK cells^46^. IFNγ is essential for STAT3 inhibitor inducing antitumor immune response^45^. In this study, STAT3 expression level was correlated with immunosuppressive genes and M2 macrophages infiltration but not T cells infiltration (Figure 3A-B). Moreover, ACT001 reduced the biomarkers of M2 macrophages and increased IFNγ and iNOS, which were probably produced by NK cells and M1 macrophages (Figure 9E-H). PD-L1 is a notorious immune checkpoint molecule. PD-L1 expression is an independent prognostic factor for poor survival in patients with various cancers^3^, ^47^-^49^. STAT3 binds to the PD-L1 promoter and it is required for the PD-L1 transcription^22^, ^50^. Inhibition and gene silencing of STAT3 resulted in decreased PD-L1 expression and restrained tumor growth^46^, ^51^. Our results confirmed that STAT3 bound to PD-L1 promoter and modulated PD-L1 transcription in glioma cells (Figure 6). Furthermore, ACT001 inhibited STAT3 phosphorylation and PD-L1 expression by directly binding to STAT3 (Figure 4 and 5).

ACT001 has a pharmacological effect similar to that of MCL and PTL. MCL is the active form released by ACT001 *in vivo*^10^ and MCL has the same α-methylene-γ-lactone group as PTL^9^. Several studies have shown that PTL has significant antitumor effects^7^, and these mechanisms can be referenced and validated for ACT001.

ACT001 is an agent that can potentially modulate antitumor immune response. PTL decreases the production of IL-6 and CCL2^52^, and MCL reduces inflammation by inhibiting the expression of IL-6, IL-10 and CCL2 in peritonitis^53^. Of note, IL-6, IL-10^54^ and CCL2^55^ play immunosuppressive roles in GBM. Furthermore, PTL^7^ and MCL^56^ are potent inhibitors of the NFκB pathway. PTL also reduces the activation of the STAT3 pathway^57^. Both the NF-κB and STAT3 pathways modulate the immune response in GBM^58^. In this study, we clarified that ACT001 reduces the transcription of PD-L1 by directly binding to STAT3. This suggests that ACT001 may have a role in reversing immunosuppression in glioma through blockade of T cell checkpoint inhibition. Immune checkpoint inhibitors have a potent ability to restore the systemic immune response^59^, ^60^ but the inability to cross the BBB limits their effects on brain glioma^61^. Given its potent ability to cross the BBB 10, ACT001 may synergize with current immunotherapies, such as anti-PD-1 therapy, to reverse the immunosuppression in glioma microenvironment.

ACT001 is a multitargeted but safe agent. PTL has an α-methylene-γ-lactone group that reacts by Michael-type addition with biological nucleophiles, especially cysteine sulfhydryl groups^62^. PTL inhibits the activation of NFκB by directly alkylating Cys38 in p65 and Cys179 in IKKβ, interferes with redox homeostasis by covalently interacting with thioredoxin and glutathione, and induces global DNA hypomethylation by alkylating the thiolate of Cys1226 in DNMT1^7^. MCL modulates tumor metabolism by selectively binding to the conserved Cys424 residue of PKM2^29^. Our data show that ACT001 directly binds to STAT3 and inhibits the activation of STAT3. This may cause concern that ACT001 may have side effects due to nonspecific targeting of healthy cells, but nonspecific attack of thiol groups is restrained, most likely due to stereochemistry and conformational changes^62^. In clinical trials, treatment is well tolerated, and no dose-limiting toxicities have occurred even at 600 mg BID^6^.

The antitumor effect of ACT001 is dose dependent. ACT001 requires a high dosage to be effective. In a mouse model of AML, 100 mg/kg of DMAMCL improves survival^56^. In a rat glioma model, 100 mg/kg DMAMCL shows a better effect than a lower dose^11^. It has been found that PTL rarely reduces tumor volumes, which is probably due to the administration of less than 100 mg/kg *in vivo*^7^. Actually, the molecular weight of ACT001 is heavier than that of DMAMCL and PTL. Our data showed that 400 mg/kg of ACT001 had a significant survival benefit in our murine glioma model, but 100 mg/kg ACT001 did not (Figure. 8). Given that ACT001 is well tolerated both in clinical trials^6^ and during long-term administration in mice^63^, we suggest that the dose of ACT001 for oral administration should not be less than 400 mg/kg in murine models.

Combined therapy may be a better strategy than monotherapy. The literature suggests that GBM may not be fully eradicated by ACT001, DMAMCL, MCL or PTL. Thus, there may be value in combining ACT001 treatment with other therapies^7^. Due to the negative immunological effects caused by systemic TMZ administration^25^, ^60^, chemotherapy in the brain may achieve a synergistic effect with ACT001. The significant decrease in PD-L1 transcription induced by ACT001 would probably contribute to reversing the immunosuppression caused by TMZ. We also hypothesize the potential beneficial effect of combining ACT001 with radiotherapy. Based on the facts that IFNγ upregulates PD-L1 expression^64^ and IFNγ expression is increased by anti-PD-1 therapy^60^, ^65^, ACT001 would probably synergize with anti-PD-1 therapy to resist the upregulation of PD-L1 expression induced by anti-PD-1 therapy.

## Conclusion

In conclusion, our work describes the molecular and clinical characterization of STAT3 and elucidates the pharmaceutical effect of ACT001 on the STAT3-PD-L1 axis. This is the first time that a direct target of ACT001 and the potential of ACT001 to modulate immunity in GBM have been described. These efforts provide the necessary evidence for clinical application and combination therapy strategies.

## Abbreviations

A: astrocytoma
AA: anaplastic astrocytoma
anti-PD-1: anti-programmed death-1 receptor antibody
AO: anaplastic oligodendroglioma
AOA: anaplastic oligodendroastrocytoma
ATCC: American type culture collection
BBB: blood-brain barrier
CCK8: cell counting kit-8 assay
CCLE: cancer cell line encyclopedia
CGGAseq: CGGA RNA sequencing
ChIP: chromatin-immunoprecipitation
CIBERSORT: cell-type identification by estimating relative subsets of RNA transcripts
DMAMCL: dimethylaminomicheliolide
DMEM: Dulbecco's modified eagle's medium
ESTIMATE: the estimation of stromal and immune cells in malignant tumors using expression data
FBS: fetal bovine serum
GBM: glioblastoma
GEO: gene expression omnibus
IF: immunofluorescence
IHC: immunohistochemistry
IVIS: *in vivo* imaging system
MCL: micheliolide
O: oligodendroglioma
OA: oligodendroastrocytoma
OS: overall survival
PD-1: programmed death receptor 1
PD-L1: programmed death-ligand 1
pGBM: primary GBM
PMA: phorbol 12-myristate 13-acetate
PTL: parthenolide
rGBM: recurrent glioblastoma
RPMI: roswell park memorial institute
RT-PCR: real-time PCR
sGBM: secondary GBM
TCGAmic: TCGA microarray
TCGAseq: TCGA RNA sequencing
TIM3: T-cell immunoglobulin mucin receptor 3
TMZ: Temozolomide
TTFields: tumor- treating fields
WHO: world health organization
