# Supplementary material for: ACT001 reduces the expression of PD-L1 by inhibiting the phosphorylation of STAT3 in glioblastoma
Source: Theranostics. 2020 May 1;10(13):5943–56. doi: 10.7150/thno.41498 (PMC7254983; doi:10.7150/thno.41498)
Supplement: Supplementary file 1 — Supplementary figures and tables. [file thnov10p5943s1.pdf]

Supplementary figures

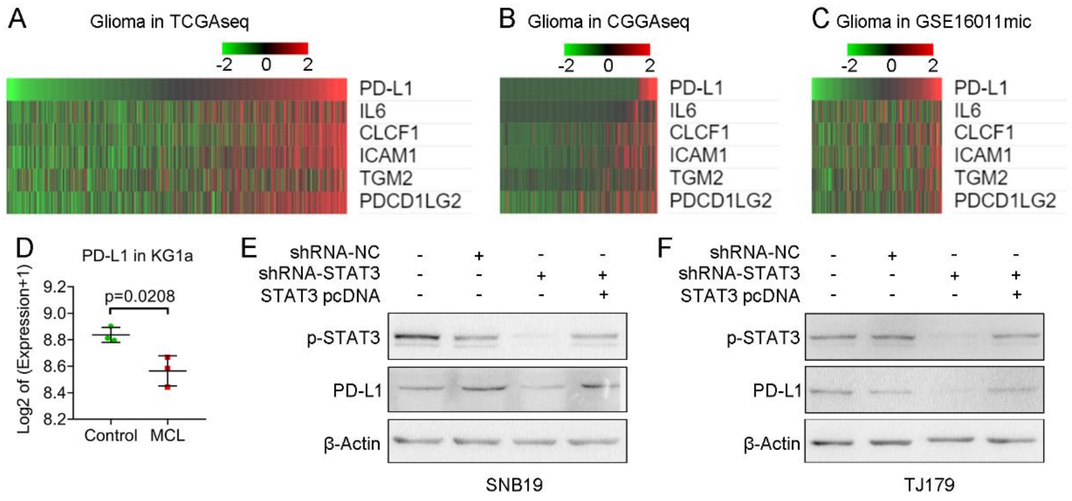

Figure S1. PD-L1 is involved in activation of STAT3 and reduced by MCL.

Heat maps correlating PD-L1 mRNA and genes related to activated STAT3 pathway (IL6, CLCF1, ICAM1, TGM2 and PDCD1LG2) in TCGAseq (A) CGGAseq (B) and GSE16011(C) datasets. Effect of MCL treatment on PD-L1 (D) transcription in KG1a cells. E, F. Proteins of PD-L1 and p-STAT3 (Tyr705) of glioma cells were detected by western blot. STAT3 pcDNA is a plasmid used to overexpress STAT3. STAT3 shRNA is a lentivirus used to knockdown STAT3.

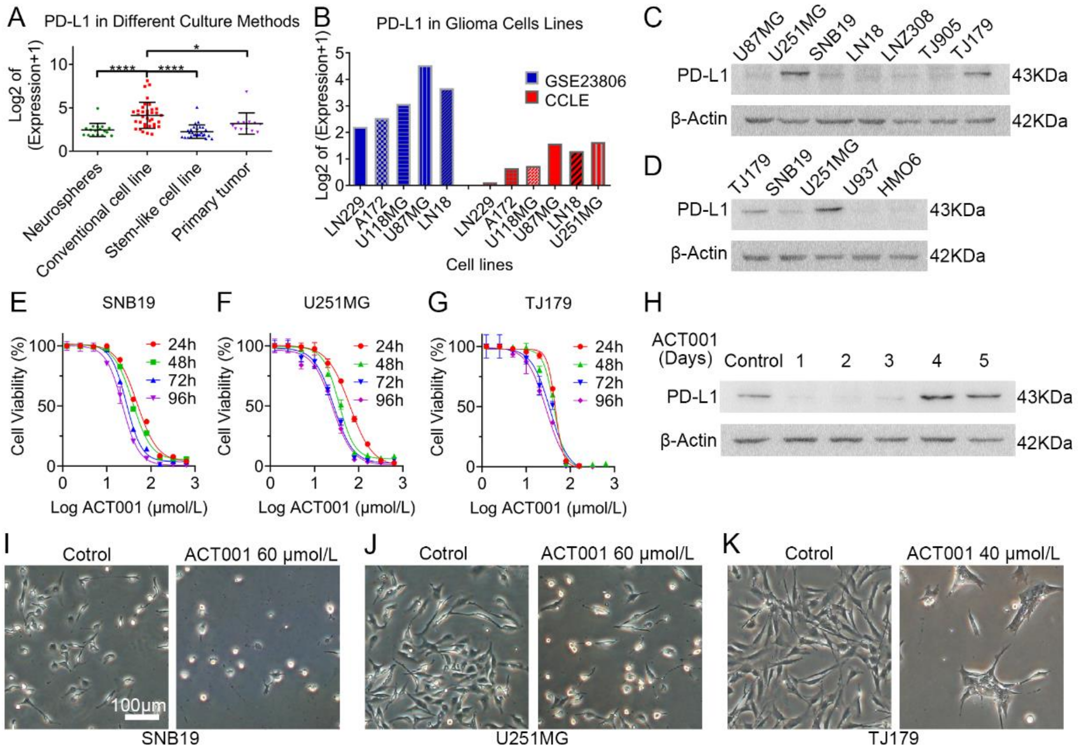

Figure S2. Consideration of cell lines and treatment of ACT001.

A. PD-L1 mRNA expression in various cell culture methods in the GSE23906 dataset. B. PD-L1 mRNA expression in various cell lines in the GSE23906 and CCLE datasets. C and D. PD-L1 protein level in glioma cell lines (U87MG, U251MG, SNB19, LN18, LNZ308, TJ905, TJ179), macrophage cell line U937, and microglia cell line HMO6. Cell viability of SNB19 (E), U251MG (F), TJ179 (G) glioma cell lines treated with ACT001 was detected by CCK8 assay. H. PD-L1 protein level in the U251MG cell line treated with 40μM ACT001 was detected at different timepoints. I-K. The morphology of glioma cells was changed by the treatment of ACT001.

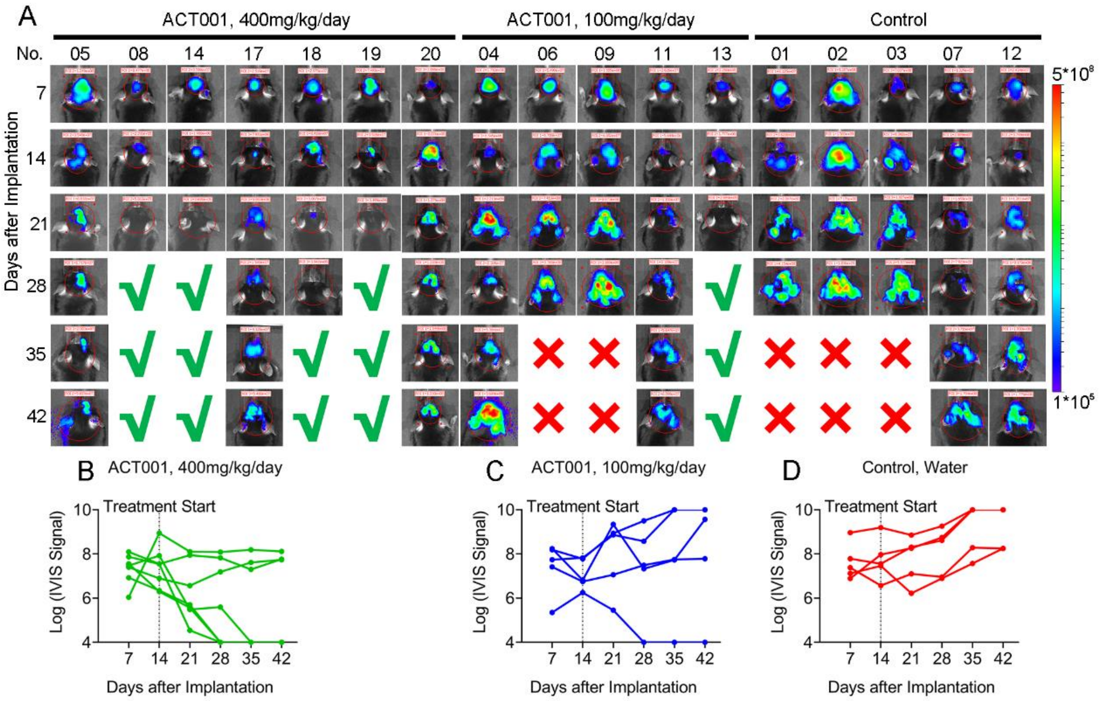

Figure S3. Progression of tumor signals in mice.

A. Luciferase imaging signal of the intracranial tumor was recorded every week for each mouse. B-D. Signal changing curve for each mouse by treatment group. Green tick, survival without tumor signal. Red cross, dead.

Supplementary methods

Pull-down of ACT001-biotin bound proteins

U251MG, SNB19 and TJ179 cells were harvested and lysed in RIPA buffer (R0010, Solarbio, China) supplemented with PMSF and protease inhibitors (P0100, dilution, 1:100; Solarbio, China) on ice for 30 min. After centrifugation at 12,000 g for 15 min at 4℃, the supernatant was collected and equally divided into three parts. The concentration of protein was measured using BCA Protein Assay Kit (PC0020, Solarbio, China) according to the manufacturer's instructions. Two of them were incubated with 100 μmol/L of ACT001-biotin and 100 μmol/L of ACT001-S-biotin in RIPA buffer overnight at 4 °C, respectively. The other one was as Input group. After incubation, added excess pre-cooled methanol and mixed, incubated in -80℃ refrigerator for 30 min to precipitated protein. After centrifugation at 12,000 g for 20 min at 4℃, washed twice with pre-cooled methanol, Next, dissolved protein pellets in phosphate buffer saline buffer (P1010, PBS; Solarbio, China) containing protease inhibitor cocktail (56383, PIC, Cell Signaling Technology, USA) and 0.1% SDS (S1010, Solarbio, China). Then, added the prewashed Streptavidin Agarose (20349, Thermo Fisher, USA) to each group and incubated for 1 h at room temperature. and the bead-bound proteins were eluted, separated by SDS-PAGE, and visualized by silver staining and western blot.

Chromatin Immunoprecipitation (ChIP) and RT-PCR

The Chromatin Immunoprecipitation (ChIP) assay was performed according to the

manufacturer's protocol (56383, SimpleChIP® Plus Sonication Chromatin IP Kit, Cell Signaling Technology, USA). We needed to prepare 1×107 cells for each cell lines. A certain amount of 37% formaldehyde (33314, Alfa Aesar, Thermo Fisher, USA) is added to make the final concentration of formaldehyde 1%, and incubated at 37℃ for 10 min. Glycine was added to terminate the cross-linking, and after mixing, it was allowed to stand at room temperature for 5 min. Then, the medium was aspirated and the cells were washed twice with ice-cold PBS and collected by a cell scraper in a 15ml centrifuge tube. After centrifugation at 1,000 g for 5 min at 4℃, the supernatant was discarded. Next, the cell lysis buffer and nuclear lysis buffer containing protease inhibitor cocktail (PIC) were added to cells orderly. Ultrasonic crushing: VCX 130 (Sonics & Materials Inc. USA), 30% power, 3s impact, 9s gap, a total of 18 min. And then centrifugated at 21,000 g for 10 min at 4℃ to remove nuclear and other debris from the sample. Inspected the effect of ultrasonication: take 50μl of ultrasonically disrupted product, added 6μl of 5M NaCl and 2μl of RNase A to incubate for 30 min, and then added 2μl of protease K and treated it at 65°C for 2 h to de-crosslinking. Purified DNA using DNA purification columns and collection tubes and detected the effect of ultrasonication. The ideal DNA fragment size is 90% chromatin fragment below 1kb, and the DNA concentration should be 50-200 μg/ml. If these conditions were met, we would proceed to the next step. In each Chromatin Immunoprecipitation (IP) reaction, 400μl of ChIP Buffer and PIC were added into 100μl of sonicated product. Next, diluted CHIP chromatin was transferred to a new EP tube with 10μl of each sample as 2% Input group. Then, added corresponding target protein antibody into each sample tube, according to the manufacturer's instructions, and mixed overnight at 4℃. Slightly re-suspended the magnetic beads and added 30μl to each IP reaction and incubated at 4℃ for 2 h. The precipitated complex was washed successively with the following solution, washing step: the solution was added and inverted at 4 ℃ for 5 min, placed the sample tube on the magnetic separation rack (7017, Cell Signaling Technology, USA) for 1-2 minutes, and carefully removed the supernatant after the solution was clarified. Washed 3 times in low salt wash buffer (CHIP buffer and DEPC water) and 1 time in high salt wash buffer (CHIP buffer, DEPC water and 5M NaCl). After washing was completed, elution was started. 150μl of CHIP elution buffer was added to each reaction sample tube, and the chromatin was eluted from the antibody/protein G microspheres by shaking and incubating at 65℃ for 30 min. Next, added 6μl of 5M NaCl and 2μl of Protease K and incubated it at 65°C for 2 h to de-crosslinking. Purified DNA using DNA purification columns and collection tubes. RT-PCR was performed to quantitative analysis of CHIP results according to the manufacturer's instructions. The primers for PD-L1 promoter used in RT-PCR as follows: forward primer, 5′- CAAGGTGCGTTCAGATGTTG -3′ and reverse primer, 5′-GGCGTTGGACTTTCCTGA- 3′.

Dual luciferase reporter gene assay

The dual luciferase reporter gene assay experiments were performed using the Dual-Luciferase Reporter Assay System (E1910, Promega, USA) according to the protocol of the manufacturer. The promoter sequence of PD-L1 (NR_052005) and sequence of STAT3 (NM_003150.4) was obtained from the NCBI web site (National Center of Biotechnology Information) and the corresponding plasmids (CD274 promoter pGL3-Basic plasmid and its negative control pGL3-Basic plasmid; STAT3 pcDNA3.1-HIS-C plasmid and its negative

control pcDNA3.1-HIS-C plasmid and pRL-TK plasmid) were prepared by Hanbio Co., Ltd. (Hanbio, Shanghai, China). 239T, U251MG and SNB19 cells (5×103 cells per well) were seeded in 96-well plates containing 100μl medium in each well. When the confluence of cells per well reached 70%, both plasmid DNA and Lipofectamine 3000 reagent were diluted in 5 μl of serum-free medium separately and incubated for 5 min. After incubation, plasmid DNA and Lipofectamine 3000 reagent were mixed gently and incubated for 15 min. Then, added them to each well containing cell and medium. Culture medium was changed after 6 h of transfection and continued to culture for 3 days. Finally, measurement of luciferase activity according to the manufacturer's protocol using Synergy 2 microplate reader (BioTek, USA).

The possible PD-L1 promoter region (2000bp ahead of PD-L1 genomic region)
>NC_000009.12:5448503-5450503 Homo sapiens chromosome 9, GRCh38.p13 Primary Assembly
CATCTGTTTTGCTTTACATATTTTTCTGAGGTAATAAAATTTCTCTTTTTCTAAACACAGCCTGTTTT
TCAATCTCCGGGTAGTTGATCAATTGTATGGGAAAATGAATGGCTGAAGGGTAGAAACAGGTG
GGAAAGATGAACAAAAACACGAATCCTCACATTACTAATACGCAAATCACTGAGCAGCAAGCT
GAGCAAATACCCTCAATTCCCATCAGCAACTTTAGAGAAAGGCAAATTCCGTTTGCCTCATTGAT
CATTTAGGTAGACCCTGAACACTGCTTTCATAAAACAAAAACAAAATACCCATCCCCAGTTTAAA
AAATTATTCATAGATCATCCAGGCCATCTAGGAGGATATGATTTAATCCTGGCTACTTGGTAAAT
TATTTGCCCAAGTTAACTCAGCTAGTTAGTGGTAGATGGCTCTGAAGCCAGTTGTTTTTTTTTGTT
TTGTTTTTTGCAGACCTCAAGAGTCATGATGAACTAGCAGATCATAAAGTTTATGCCCTGGGTCT
TGACCATTTTTAGAAAAATAAAACATTAAATGAAAATATCAGAGGGCATTGCAGATAGTAGATCT
AAGTATTTTTTCATGAAACTTGTTGTACATGTGTGTGTCATACACAGACTATATATATGCAGTACC
TGTAAACTGTATTGCCACATAATGTCTATATTTTCCTAGAGGTCACAGTCACCAAAGTTGGGAAG
TCACCCAACTTCGGGAACTTTGGGAAGTCACCCAAACTTACAGTCACCAAAATTGCTCTATTCTA
CTATGTGACCTCAAAAGTGATTTGAAAGAAGGAACATCTGAGCTGGGCCCAAACCCTATTGCAA
TTTTATTGGGGCCAAAGAGAACTCCATGCTCCTGCCAAATCAAGGCAGTGTCAGCCTCAATAAT
TTCCCAGATAAAAATAAAAATCTGTGATACAATCAGAATGTGAAAATTCTTATTTTGGAAGCAAA
TGTCATAACCAATGCAAGGGCTATCTCAATATTCATTCATTATGCAGTATTTTGAACTGCAGTTGA
AATGAATAAGAAGGAAAGGCAAACAACGAAGAGTCCAATTTCTCAATTTAGAAAAAGAGAAAA
AAAAGAAAAGGGAGCACACAGGCACGGTGGCTCAAGCCTGTAATATCAGCACTTTGGCGGATC
ACTTGAGGTCAAGGAGTTCGAGAAAAGAGAGCACCTAGAAGTTCAGCGCGGGATAATACTTAA
GTAAATTATGACACCATCGTCTGTCATCTTGGGCCCATTCACTAACCCAAAGCTTTCAAAAGGGC
TTTCTTAACCCTCACCTAGAATAGGCTTCCGCAGCCTTAATCCTTAGGGTGGCAGAATATCAGGG
ACCCTGAGCATTCTTAAAAGATGTAGCTCGGGATGGGAAGTTCTTTTAATGACAAAGCAAATGA
AGTTTCATTATGTCGAGGAACTTTGAGGAAGTCACAGAATCCACGATTTAAAAATATATTTCCTA
TTATACACCCATACACACACACACACACCTACTTTCTAGAATAAAAACCAAAGCCATATGGGTC
TGCTGCTGACTTTTTATATGTTGTAGAGTTATATCAAGTTATGTCAAGATGTTCAGTCACCTTGAA
GAGGCTTTTATCAGAAAGGGGGACGCCTTTCTGATAAAGGTTAAGGGGTAACCTTAAGCTCTTA
CCCCTCTGAAGGTAAAATCAAGGTGCGTTCAGATGTTGGCTTGTTGTAAATTTCTTTTTTTATTAA
TAACATACTAAATGTGGATTTGCTTTAATCTTCGAAACTCTTCCCGGTGAAAATCTCATTTACAAG
AAAACTGGACTGACATGTTTCACTTTCTGTTTCATTTCTATACACAGCTTTATTCCTAGGACACCA
ACACTAGATACCTAAACTGAAAGCTTCCGCCGATTTCACCGAAGGTCAGGAAAGTCCAACGCC
CGGCAAACTGGATTTGCTGCCTTGGGCAGAGGTGGGCGGACCCCGCCTCCGGGCCTG
